# Oxidation of Wine Polyphenols by Electrochemical Means in the Presence of Glutathione

**DOI:** 10.3390/antiox12101891

**Published:** 2023-10-21

**Authors:** Emad F. Newair, Abdulaziz Al-Anazi, François Garcia

**Affiliations:** 1 Sciences for Oenology (SPO), Montpellier University, National Research Institute for Agriculture, Food and the Environment (INRAE), Institut Agro, F-34060 Montpellier, France; 2 Department of Chemical Engineering, College of Engineering, King Saud University, P.O. Box 800, Riyadh 11421, Saudi Arabia

**Keywords:** wine polyphenols, glutathione, electro-oxidation, cyclic voltammetry, oxidation mechanism

## Abstract

The oxidation of wine may be beneficial or harmful to its quality. On the one hand, controlled oxidation can lead to the development of desirable sensory characteristics for red wine, such as enhanced color stability. Alternatively, oxidation can lead to white wine browning and a decrease in fruity aromas, and the development of an off flavor and wine polyphenols are also involved. The presence of glutathione (GSH) can help mitigate the negative effects of oxidation by acting as a protective antioxidant. In order to better understand the antioxidant role played by GSH, wine polyphenols oxidation experiments by electrochemical means in the presence of GSH were carried out. The oxidation behavior of polyphenols representing different phenolic classes commonly found in wines, including protocatechuic acid (PCA), caffeic acid (CAF), epicatechin (EC), and rutin (Ru), was investigated using cyclic voltammetry and bulk electrolysis. We identified the oxidation products and reaction pathways of these polyphenols using ultra-high-performance liquid chromatography coupled with mass spectrometry (UPLC-MS), in both the absence and the presence of glutathione (GSH). UPLC-MS was utilized to demonstrate that, in the presence of glutathione (GSH), the four molecules were subjected to electrochemical oxidation, resulting in the formation of mono- and bi-glutathione conjugates. A two-electron oxidation process combined with the removal of two protons is the first step in transforming polyphenol molecules. As a result, the corresponding quinone is formed. The quinone can then be reduced back to its original form by glutathione (GSH), or it can interact further with GSH to produce mono- and bi-glutathione conjugates. These results contribute to understanding and predicting the oxidative degradation pathway of polyphenols in wine. Understanding this process seems important for winemakers to control and optimize the sensory characteristics of their wines.

## 1. Introduction

White wines are highly susceptible to oxidation reactions [1]. As a result, the color can brown, and the aroma and flavor of a particular varietal can disappear. Extensive studies were focused on the chemical process concerning wine oxidation [2]. In spite of the fact that many questions remain unanswered regarding non-enzymatic wine oxidation, it remains unclear how it occurs [3]. The initial step in the oxidation process of wine concerns phenolic compounds [4], and their oxidation is catalyzed by transition metal ions such as copper and iron [5]. As a result of the process, a semiquinone radical is formed [5], which is afterward oxidized in quinone. It is likewise possible to oxidize polyphenols by the hydroperoxyl radical [6,7], which occurs because of oxygen reduction. During the pressing and bottling process of white wines, SO_2_ is added to prevent must and wine oxidation. SO_2_ plays a crucial role in the handling and storage of wine. Its presence effectively prevents the occurrence of browning [8], which can negatively impact the quality and appearance of the wine. Additionally, SO_2_ acts as a stabilizer by slowing down the degradation of esters [9], which are responsible for the fruity aroma in wines. Moreover, SO_2_ helps to preserve the varietal thiols [10], which contribute to the unique and distinctive flavors of different wine varieties. Certain groups of people may, however, be sensitive to SO_2_ and may experience allergic reactions, including dizziness, abdominal pain, and headaches. An acceptable daily sulfite intake for humans of 0.7 mg/kg body weight has been determined by the FAO/WHO expert committee concerning food additives [11]. Consequently, there has been an intensifying inclination these last years to restrict SO_2_ utilization [12,13] and also to explore substitute options. This trend reflects the growing awareness of the potential health implications associated with sulfite intake and the desire to minimize its presence in various food products. The pursuit of alternatives to SO_2_ is driven by the objective of ensuring food safety and promoting the well-being of individuals.

Grape phenols can undergo oxidation, which is facilitated by polyphenol oxidase enzymes (PPO) during the pressing process. Furthermore, *o*-quinones are formed through the chemical oxidation of *o*-diphenols during the aging process. It has been observed that the quantity of *o*-diphenols present in white wines is directly proportional to the browning degree that occurs. This browning is a consequence of enzymatic oxidation leading to the formation of polymerized *o*-quinones [8,14,15]. As a powerful antioxidant, glutathione (GSH) reduces the oxidative loss of aroma and the browning of white wine. During the winemaking process, it appears crucial to comprehend the significant role of GSH in the limitation of phenol oxidation. GSH can react with caftaric acid, resulting in 2-S-glutathionyl caftaric acid formation, which is commonly mentioned as Grape Reaction Product (GRP). This reaction is of utmost importance as it helps to control and prevent the oxidation of phenols in wine production. By reacting with caftaric acid, GSH effectively safeguards the phenolic compounds which are in grapes, ensuring the preservation of their desired characteristics during the winemaking process. The formation of GRP serves as a protective mechanism, contributing to the quality and stability of the produced wine [16]. According to the research findings [17,18], it has been observed that GSH can exert a beneficial influence on the color of white wine. The color appears to become more stable during wine aging, which can be attributed to the presence of GSH. These findings suggest that GSH is of great importance in maintaining a satisfying white wine color over time. There is clear evidence that GSH has a protective effect on specific wine aromas. Because it competes for *o*-quinones, it may cause a decrease in *o*-quinone-thiol associations, resulting in a more important part of thiol-related aromas in wine. The preservation of aroma compounds in bottles during storage is a well-known effect of GSH. Specifically, GSH has been found to effectively preserve compounds such as isoamyl acetate, ethyl hexanoate, and linalool [18]. This preservation ability is crucial for maintaining the aromatic qualities of the stored substances over an extended period. When caffeic acid is in wine at specific concentrations, a protective effect of GSH appears to be remarkably efficient [12]. The concentration of GSH in grapes can surpass 100 mg kg^−1^, and this may vary depending on the function of the grape cultivar, environmental conditions, and viticultural practices [19]. GSH concentrations in grape juice can vary between 10 and 100 mg L^−1^. Moreover, exposure to oxygen, tyrosinase activity, and the maceration of grape skin during the pre-fermentation process may influence the GSH level in grape juice [20,21]. However, wine generally contains a lower GSH concentration compared to grape juice and grapes, typically between 1 and 20 mg L^−1^ [22]. White wines that have a concentration of glutathione above 6–10 mg L^−1^ have been observed to exhibit superior preservation of color and aroma during aging and storage [23]. In wine, the GSH levels can also depend on the presence of Saccharomyces cerevisiae during the fermentation process and less aging, as mentioned in reference [24]. Moreover, the International Organization of Vine and Wine (OIV) authorizes the GSH addition in wine or must at a maximal concentration of 20 mg L^−1^.

Glutathione is the most profuse non-protein sulfhydryl compound in cells [25] and is generally regarded as cytoprotective when it is combined with quinones. Its thiol group helps as a “sacrificial” nucleophile, preventing irreversible alterations to important nucleophilic sites on macromolecules. By acting as a sacrificial agent, GSH effectively shields and preserves these critical sites, ensuring their functionality and integrity. It must be signaled that substitute effects notably influence the redox potential of quinone [26,27]. As a consequence, when an electronegative substitute is added to a quinone, the oxidant becomes stronger, and the reduced or hydroquinone form becomes less susceptible to oxidation. The electron-donor groups added to a quinone often lead to a decrease in its oxidizing power, resulting in hydroquinone formation, which may be more willingly oxidized. This is due to the influence of these groups. Consequently, when a nucleophile is added to a quinone, the hydroquinone that is formed can typically be reoxidized by the original, unsubstituted quinone [28]. Therefore, a single quinone can perform multiple nucleophilic additions [29,30], or it can form cross-links between nucleophilic sites on macromolecules [31]. Wine polyphenols can undergo oxidation when exposed to a glassy carbon electrode [32]. This characteristic enables the utilization of electrochemical techniques to deliberately produce quinones [33]. Subsequently, these quinones can be examined in conjunction with other wine components, such as glutathione, allowing for further study and analysis. Some authors have formulated diagnostic criteria to assess different electrode mechanisms [34,35]. These criteria are commonly utilized in cyclic voltammetry experiments that aim to explore the reactions of quinones generated by electrochemical means with nucleophiles [36,37,38,39,40,41]. These proposed diagnostic criteria provide a valuable framework for analyzing and understanding the complex electrochemical processes involved in these reactions. By employing these criteria, researchers can gain insights into the underlying mechanisms and kinetics of quinone–nucleophile interactions, thereby contributing to advancements in the field of electrochemistry.

In this paper, we will delve into the electrochemical interactions between glutathione and polyphenols (protocatechuic acid (PCA), caffeic acid (CAF), epicatechin (EC), and rutin (Ru)) as well as the characterization of the resulting oxidized products by ultra-high-performance liquid chromatography-mass spectrometry (UHPLC-MS). The four compounds listed above represent different phenolic classes commonly found in wines. In terms of chemical structure, PCA belongs to the class of hydroxybenzoic acids, whereas CAF belongs to the class of hydroxycinnamic acids. EC belongs to the flavan-3-ols class, also known as catechins, and Ru (used as quercetin glycoside) belongs to the flavonols class commonly found in wines. In terms of sensory profile and health properties, these compounds represent different classes, each with its own particular characteristics. Understanding these interactions is crucial for comprehending the chemical changes that occur in wine and their possible influence on its quality as well as health benefits. The current technique (EC/UHPLC-MS) allows the separation and identification of numerous compounds present in complex mixtures such as wine. By analyzing the mass-to-charge ratios and fragmentation patterns of the oxidized products, valuable insights can be gained into their chemical structures and potential biological activities. The use of UHPLC-MS to study glutathione-polyphenols of wine interactions enables researchers to identify specific oxidation products. This information can help elucidate the underlying chemical processes and shed light on the influence of these interactions on wine concerning its quality and stability as well as health benefits.

## 2. Materials and Methods

### 2.1. Chemicals

Chemical compounds were purchased from reputable suppliers. Protocatechuic acid (PCA) was obtained in its pure form with a purity level of 100%. Caffeic acid (CAF) was purchased with a minimum purity of 98.0%. Epicatechin (EC) was acquired with a purity of at least 98.0% (by HPLC determination). Rutin hydrate, another compound used in the study, was obtained at 94.0% purity by HPLC determination. Sodium hydroxide was purchased with a minimum purity of 98% from Sigma–Aldrich (Saint-Quentin-Fallavier, France). Ethanol, with a purity of 99.8% as determined by HPLC, was provided by Merck (Rahway, NJ, USA). Lastly, alumina powder was supplied by Metrohm (Villebon-sur-Yvette, France). These high-quality materials were chosen to ensure accurate and reliable results in the experiment. Solutions were made with purified water (Millipore (Milli-Q) system, St. Louis, MI, USA).

### 2.2. Solutions and Sample Preparation

A stock solution of each of the four chemical compounds, PCA, CAF, EC, and Ru, was prepared before the experiment. The concentration of each stock solution was 1.0 × 10^−3^ M. To prepare these solutions, an ethanol/water mixture in a ratio of 50:50 was used as the solvent. In addition to the stock solutions, a synthetic wine was used as the supporting electrolyte containing ethanol (12% (*v*/*v*)) and L-tartaric acid with a concentration of 33 mM and pH of 3.6 adjusted using 1 M NaOH. To ensure the stability of the prepared solutions, they were stored in a refrigerator. Moreover, to protect them from light-induced degradation, aluminum foil was used to cover the containers. These precautions were taken to maintain the integrity and reliability of the solutions throughout the experiment. To carry out each new experiment, fresh solutions were dispensed from stock solutions. This process of dilution was performed to ensure that the solutions used in the experiments were prepared specifically for each instance.

### 2.3. Instruments

The Autolab Potentiostat/Galvanostat (model PGSTAT302N), in conjunction with the NOVA 2.0 software developed by Eco-Chemie in Utrecht (The Netherlands), was employed to carry out the electrochemical measurements. These measurements were conducted using a conventional three-electrodes electrochemical cell containing 20 mL of solution. In this work, various electrodes were utilized for different purposes. The working electrode employed was a 3.0 mm diameter electrode made of glassy carbon. This specific glassy carbon electrode (GCE) model numbered 61204300 was obtained from Metrohm-Autolab in Switzerland. A silver/silver chloride (Ag/AgCl) electrode was employed as a reference electrode. The Ag/AgCl electrode remained immersed in an aqueous solution of potassium chloride (aq. KCl) with a concentration of 3.0 M. This reference electrode served as a standard for the working electrode potential measurement. Lastly, an auxiliary electrode made of platinum wire was used. The primary function of this electrode was to provide electrical contact and complete the circuit. By utilizing these three electrodes, the experiment was able to measure and analyze the electrochemical reactions accurately.

The GCE surface underwent a cleaning process before each measurement. This involved polishing with alumina powder (0.3 μm), followed by thorough rinsing with purified water. Then, the GCE was placed in an ultrasonic bath for 5 min. An electrochemical cleaning was applied to the GCE after the mechanical polishing in the model wine solution by cyclic voltammetry until it reached a steady state. It is worth noting that all measurements were conducted in triplicate to ensure accuracy and reliability.

To measure the pH of the solution, a bench-top pH meter with the model number HI 2210, produced by HANNA Instruments in Romania, was employed. The pH meter featured a combined pH reference electrode, which allowed for accurate and precise pH measurements of the solution throughout the experiment.

### 2.4. Electrolysis

The electrolysis reaction cell utilized in this study was the µ-PrepCell, manufactured by Antec in the United States. This cell consisted of three main components: a working electrode, made of glassy carbon with a surface area of 1.9 cm^2^, a counter electrode made of titanium, and a reference electrode with a Pd/H_2_ composition (HyREF). In the process of electrochemical oxidation, the objective analyte was dissolved until it reached a concentration of 0.1 × 10^−3^ M in the model wine. The solution of the analyte (at a concentration of 0.1 × 10^−3^ M) was then consistently circulated in the electrochemical cell with a flow rate equal to 0.1 mL/min. Throughout this process, a fixed potential was continuously maintained between the GCE and the HyREF. The baseline stabilization was achieved by maintaining a constant potential for a duration exceeding 5 min. Following this, the samples were collected in vials that had been dyed and placed under an argon atmosphere. These samples were then subjected to LC-MS analysis.

### 2.5. UPLC-DAD-MS System

An Acquity UPLC, manufactured by Waters in Milford, MA, was employed in conjunction with a mass spectrometer to detect and classify wine polyphenols, as well as the glutathione conjugates derived from these polyphenols. To carry out the liquid chromatographic analysis, the Acquity UPLC was outfitted with a photodiode array detector. A column made of High-Speed Steel (HSS T3) with dimensions of 100 × 2.1 mm and a 1.8 mm column was used in this experiment. The specific column used was Nucleosil 120-3 C18 end-capped, produced by Macherey-Nagel in Sweden. For the chromatographic separation, the gradient conditions were as follows: Solvent A consisted of water/formic acid in a ratio of 99:1 (*v*/*v*). Solvent B was acetonitrile/water/formic acid in a ratio of 80:19:1 (*v*/*v*/*v*). The initial composition of the mobile phase was 0.1% B. From 0 to 5 min, there was a linear increase in the proportion of solvent B, reaching 60% B at 5 min. From 5 to 7 min, the proportion of solvent B was further increased to 99%, and this composition was maintained isocratically for the remainder of the analysis. These gradient conditions were used to achieve the desired separation and elution of the target compounds in the sample. The online coupling of the Acquity UPLC system with the amaZon X ESI-Trap mass spectrometer from Bruker Daltonics in Bremen, Germany was established. The nebulizer pressure during analysis was maintained at 44 psi, while the dry gas temperature was 200 °C and its flow rate was 12 L min^−1^. For optimal performance, a capillary voltage of 4 kV was applied. These conditions were carefully chosen to ensure efficient and accurate analysis of the samples. The mass spectrum data were obtained within the range of 90–1500 Th. At last, an acquisition speed of 8.1 *m*/*z* min^−1^ for the mass spectrum was applied.

## 3. Results and Discussion

### 3.1. Cyclic Voltammetry of Polyphenols (PCA, CAF, EC, and Ru) in the Absence of Glutathione

**Figure 1:**
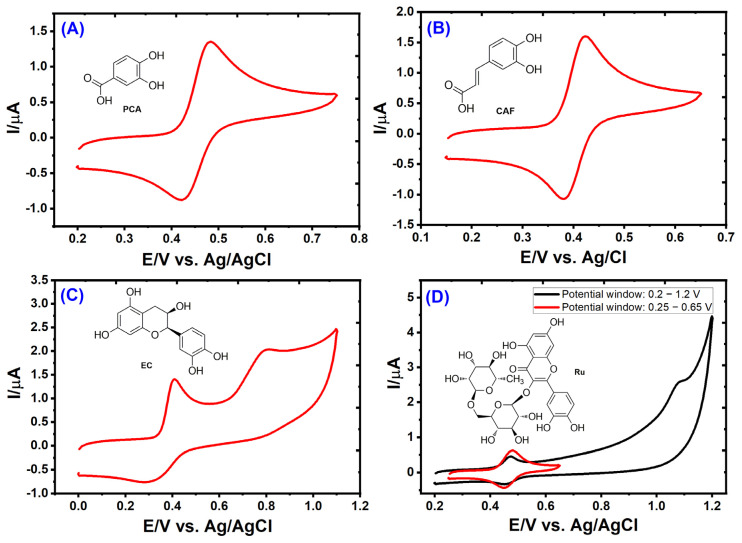
Cyclic voltammograms of (**A**) 100 µM PCA, (**B**) 100 µM CAF, (**C**) 100 µM EC, and (**D**) 100 µM Ru in synthetic wine (pH 3.6) on GCE at a scan rate of 20 mV s^−1^ and without GSH.

**Scheme 1:**
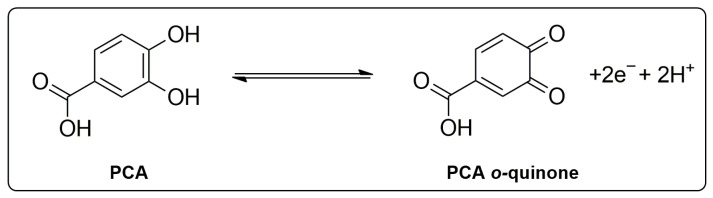
The oxidation mechanism of PCA.

Cyclic voltammograms were conducted on a GCE in a synthetic wine (pH of 3.6) at 20 mV s^−1^ for scan rate. The voltammograms were obtained at a concentration of 100 µM for the four studied compounds: PCA, CAF, EC, and Ru. The voltammograms are presented in Figure 1A–D. The obtained cyclic voltammogram revealed interesting electrochemical features. PCA exhibits an oxidation peak at a potential of 0.48 V in Figure 1A. This peak represents the oxidation of PCA to its corresponding oxidized form (i.e., *o-*quinone form). A reduction peak was noticed for a potential of 0.42 V, illustrating the conversion of the *o-*quinone form back to PCA. The presence of these oxidation and reduction peaks (ΔE_p_ = 60 mV vs. Ag/AgCl) in the cyclic voltammograms suggests that PCA goes through a quasi-reversible redox process for the applied potential range. This redox process is due to hydroxyl groups in PCA that can readily donate electrons to form the *o-*quinone according to Scheme 1 [42]. Figure 1B presents the cyclic voltammogram of a 100 µM CAF solution in a model wine solution with a pH of 3.6. The voltammogram showed an anodic peak at 0.424 V as well as a cathodic one at 0.383 V. These findings provide valuable insights into the electrochemical behavior of CAF in synthetic wine. Moreover, no further anodic or cathodic reaction was detected at higher or lower potentials. As a result of the reversible redox couple of the CAF, two electrons and two protons are transferred, resulting in the generation of *o-*quinones. It is important to mention that the ΔE_p_ value is 41 mV, which is higher than 30 mV, the expected value corresponding to a reversible two-electron transfer. This discrepancy implies a limitation that stems from the charge kinetics, indicating the presence of a quasi-reversible process. The obtained findings align with a previously published study on the oxidation of CAF, indicating that the catechol group undergoes a two-electron oxidation process [43]. Throughout a sequence of irreversible chemical reactions at each stage, the ultimate outcome is the formation of *o*-quinone. These outcomes are consistent with the conclusions drawn in the aforementioned report.

During the cyclic voltammetry analysis of EC, two distinct oxidation waves were observed (Figure 1C). The first oxidation wave occurred at a relatively low potential E_p_ = 0.41 V vs. Ag/AgCl, indicating the catechol moiety oxidation into *o-*quinone. The low potential oxidation wave observed can be explained by the extraction of two electrons from the phenolic hydroxyl groups located in the epicatechin B ring. The A ring resorcinol moiety undergoes oxidation on a second wave, which is observed at a higher potential of E_p_ = 0.80 V vs. Ag/AgCl. This oxidation process involves the removal of additional electrons via an irreversible redox reaction. In the cyclic voltammogram, two oxidation waves suggest a multi-step oxidation mechanism for EC. The involvement of phenolic hydroxyl groups and the formation of a quinone-like species indicate the importance of redox reactions and electron transfer processes in the oxidation of EC. Additionally, as shown in Figure 1C, the initial anodic peak was detected at 0.41 V, while a subsequent cathodic peak was obtained at a potential of 0.30 V. As a result of the reversible redox couple concerning EC, two electrons and two protons are transferred, resulting in the generation of *o-*quinones. In addition, it is noteworthy that the ΔE_p_ is 110 mV, suggesting an irreversible process. Thus, the behavior exhibited in this study is reminiscent of the oxidation behavior observed in earlier studies of the epicatechin monomer [44].

The cyclic voltammetry (CV) of 100 µM Ru in synthetic wine on the glassy carbon electrode was recorded between +0.2 and +1.2 V (Figure 1D, black voltammogram). The voltammogram revealed the presence of a single well-defined oxidation peak at a potential of 0.41 V (labeled peak 1) along with a shoulder at 1.084 V. Thus, the oxidation peak signifies *o-*quinone formation using the oxidation process (−2e^−^/2H^+^) of the B ring catechol group. On the other hand, the oxidation shoulder corresponds to the hydroxyl group’s oxidation in the less electroactive ring A [45]. It has been previously documented that the catechol B-ring hydroxyl groups present are more susceptible to oxidation compared to those in the resorcinol A-ring [46,47]. These findings align with the current observations, indicating a consistent pattern across studies. Peak 1 is observed to be in perfect agreement with the reversible reaction when a potential scan switch to +0.65 V is performed. Within the potential range of 0.25 to 0.65 V, a distinct redox wave is obtained for Ru (as shown in Figure 1D, red voltammogram). The anodic peak potential (E_p_^a^) was measured to be 0.47 V, while the cathodic peak potential (E_p_^c^) was determined to be 0.43 V. The separation between the peak potentials, ΔE_p_, was determined to be 40 mV. This value is slightly more important than the expected value related to a two-electrons fully reversible system, which is 59/n mV [48]. Additionally, the peak currents quotient (I^c^/I^a^) was found to be less than unity, suggesting that the oxidation-reduction process of Ru is quasi-reversible at GCE.

### 3.2. Cyclic Voltammetry of Polyphenols (PCA, CAF, EC, and Ru) in the Presence of GSH

**Figure 2:**
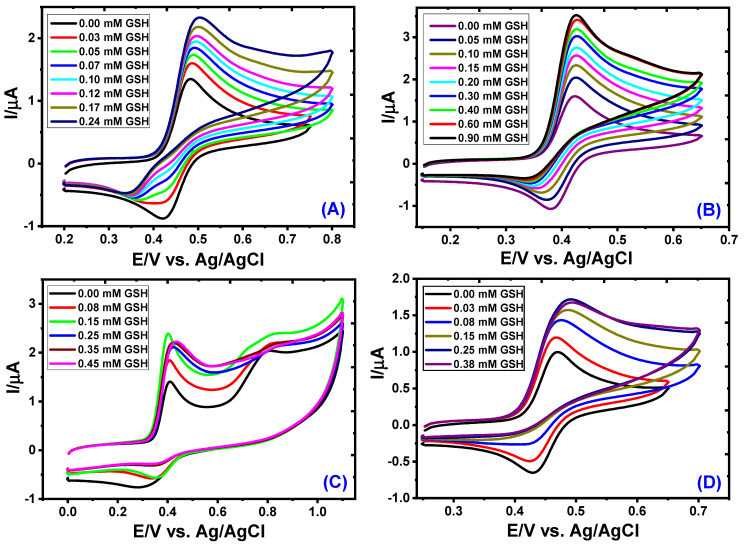
Cyclic voltammograms of (**A**) 100 µM PCA, (**B**) 100 µM CAF, (**C**) 100 µM EC, and (**D**) 100 µM Ru in synthetic wine at pH 3.6 on GCE at a scan rate of 20 mV s^−1^ when different amounts of GSH are added.

An investigation of the glutathione effect on cyclic voltammograms of PCA, CAF, EC, and Ru was carried out in synthetic wine at pH 3.6 on GCE which was used due to its excellent conductivity and stability (Figure 2). As shown on voltammograms obtained when GSH is present in solution, interesting changes in oxidation and reduction occurred. As a result of the GSH addition, the oxidation current increased. Based on these observations, GSH may act as a redox mediator or promote electron transfer during oxidation. A possible explanation for the increased oxidation current is the interaction between polyphenols and GSH, which may alter polyphenols’ redox behavior. In contrast, GSH decreased the peak current for polyphenols’ *o-*quinone reduction. Based on the decline in reduction currents, it appears that GSH is inhibiting the reduction process or competing with quinones for electrons. As a result, the presence of GSH leads to a decrease in the formation of quinone species [49]. According to the findings, it seems that when GSH is present, there is an involvement in the oxidation of polyphenols. In this case, electrons transfer in a quasi-reversible way, and chemical reactions occur irreversibly. In this step, the transfer of electrons occurs to form the corresponding *o-*quinone. In addition to that, an irreversible chemical reaction occurs between *o-*quinone and GSH, illustrated by the diminution of the peak currents as well as the current ratio. The voltammograms demonstrate that the electro-catalytic activity of *o*-quinones facilitates the reaction between thiols and quinone, resulting in a swift interaction between quinone and glutathione [50]. This observation suggests that *o*-quinones play a significant role in mediating the interaction between thiols and glutathione, potentially influencing various biochemical processes. The rapid interaction between quinone and glutathione highlights the importance of understanding the electrochemical properties of *o*-quinones and their impact on thiol-related reactions. As a result of these findings, we gain insight into the redox behavior of polyphenols when GSH is present in wine systems.

**Figure 3:**
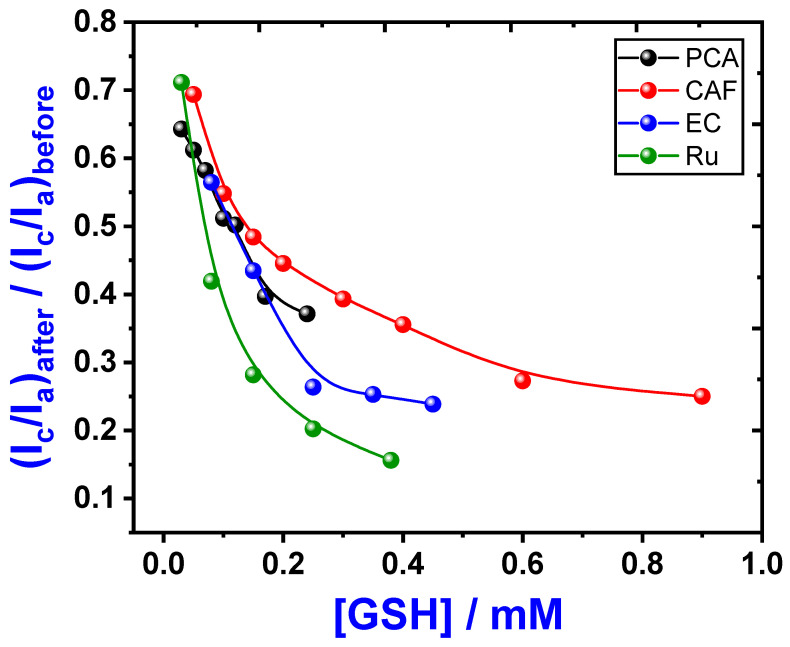
Cathodic peak current to anodic peak current ratios (extracted from Figure 2), before and after GSH additions, in synthetic wine at pH 3.6 with 100 µM of each PCA, CAF, EC, and Ru.

The evaluation of interactions between polyphenols and glutathione can be determined by introducing varying glutathione concentrations into the polyphenol solutions. Indeed, in this way, researchers can assess the extent of the interactions between these two compounds [33]. This method allows for the quantification and analysis of the interaction rate, providing valuable insights into the biochemical processes involving GSH and polyphenols quinone. The anodic peak current, denoted as I^a^, exhibited a direct relationship with the amount of polyphenol present that was ready to undergo oxidation, leading to quinone form formation. On the other hand, the cathodic peak current, represented as I^c^, demonstrated a proportional dependence on the quantity of quinone available for reduction back to its original polyphenol state. Thus, the extent of interaction between quinone and GSH for the experiment can be determined by analyzing the diminution of the ratio I^c^/I^a^ after the addition of GSH, as compared to the ratio before. This decrease provides evidence for the extent to which GSH interacts with quinone in the experimental setup (Figure 3). By examining this change in ratio, researchers can gain insights into the nature and strength of the interaction between GSH and quinone. The interaction between Ru and GSH was found to be more pronounced compared to the interaction between GSH and other polyphenols. This suggests that Ru quinone moiety exhibits a rapid interaction with GSH. Moreover, the I^c^/I^a^ ratio shows a significant decrease, which indicates that GSH plays a role in stabilizing Ru when it is exposed to oxidative stress. In contrast, when glutathione is added to CAF a slight decrease in the I^c^/I^a^ ratio occurs. This reduction suggests that the CAF quinone form had minimal interaction with glutathione. These findings indicate that GSH may provide a protective effect against oxidation for polyphenols, albeit to varying degrees. The ability of GSH to prevent polyphenols from undergoing oxidation highlights its potential role as an antioxidant in biological systems.

### 3.3. Bulk-Electrolysis Flow-Cell System for the Oxidation of Polyphenols with Added GSH and Oxidation Products Characterization Using UHPLC-MS

A series of additional experiments were conducted utilizing a commercially available coulometric cell in conjunction with LC-MS. The goal of these experiments remained to study the electrochemical oxidation behavior of the studied polyphenols (PCA, CAF, EC, and Ru) in the presence of GSH at a pH of 3.6. This part sought to gain insights into the chemical reactions and transformations that occur under these specific conditions. The combination of the coulometric cell and LC-MS allowed for an accurate analysis and characterization of the oxidation behavior of the studied polyphenols. Two ways were employed to achieve the desired outcome. First, the focus was on collecting the oxidized polyphenols that passed through the electrochemical cell (method 1). To avoid the direct electro-oxidation of GSH, these polyphenols were directly collected in a solution containing 1.0 mM GSH. This approach aimed to preserve the integrity of GSH while efficiently collecting the oxidized polyphenols. Secondly, the objective was to use the polyphenol solution when GSH is present in excess (1.0 mM) and then flow it through the cell of electrolysis (method 2). This approach ensured that there was an ample amount of GSH available to react with the polyphenols, facilitating the desired reactions within the electrolysis cell [51]. By employing these two distinct methods, it aimed to explore different approaches for handling oxidized polyphenols and GSH in the electrochemical cell. Each method offered unique advantages and considerations, and their implementation allowed for a comprehensive investigation of the desired reactions. After the application of the potential, it was observed that a notable portion of glutathione still existed in its reduced form following electrolysis, as shown by UPLC-MS data, even though the GSH was directly oxidized at the electrode surface. This indicates that the reduced GSH had the potential to interact with quinone species, as supported by previous research [33]. For the two approaches, it was acquired significant quantities of adduct products. Subsequently, the solutions after electrolysis underwent examination utilizing UPLC-MS to determine the principal conjugated species generated when GSH was present.

**Figure 4:**
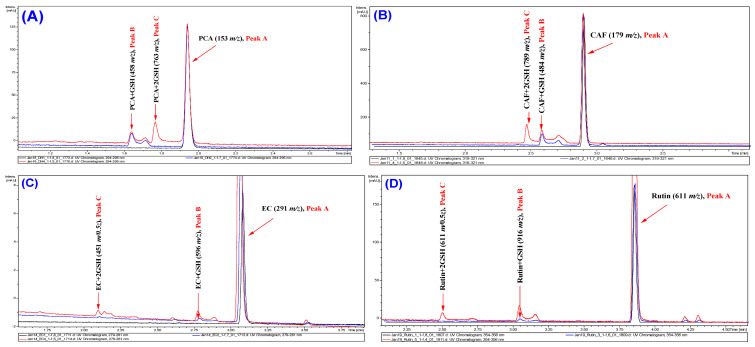
UPLC–UV chromatograms of (**A**) 100 µM PCA solution (black line), 100 µM PCA solution oxidized at 300 mV vs. Pd/H_2_ and trapped by a 1.0 mM GSH solution (blue line) and 100 µM PCA solution oxidized at 300 mV vs. Pd/H_2_ in presence of 1.0 mM GSH solution (red line) at 295 nm, (**B**) 100 µM CAF solution (black line), 100 µM CAF solution oxidized at 220 mV vs. Pd/H_2_ and trapped by a 1.0 mM GSH solution (blue line) and 100 µM CAF solution oxidized at 220 mV vs. Pd/H_2_ in presence of 1.0 mM GSH solution (red line) at 320 nm, (**C**) 100 µM EC solution (black line), 100 µM CAF solution oxidized at 200 mV vs. Pd/H_2_ and trapped by a 1.0 mM GSH solution (blue line) and 100 µM CAF solution oxidized at 200 mV vs. Pd/H_2_ in presence of 1.0 mM GSH solution (red line) at 280 nm, and (**D**) 100 µM Ru solution (black line), 100 µM Ru solution oxidized at 300 mV vs. Pd/H_2_ and trapped by a 1.0 mM GSH solution (blue line) and 100 µM Ru solution oxidized at 300 mV vs. Pd/H_2_ in presence of 1.0 mM GSH solution (red line) at 355 nm.

In the analysis of the PCA solution after electrolysis, it was found that there was no occurrence of electrochemical oxidation of polyphenols when the working electrode potential was maintained at 0 V (vs. Pd/H_2_). Instead, just one single peak corresponding to *m*/*z* 153 [M–H]^−^ was observed. This peak can be attributed to PCA, as depicted in Figure 4A by the black line, specifically labeled as peak A. A novel product was observed at an applied potential of 0.3 V. This detection was achieved by capturing the electrolyzed solution when glutathione was utilized as a trapping agent, employing method 1. The presence of this new product is indicated by the blue line in Figure 4A, specifically at peak B. A significant signal observed at *m*/*z* 458 [M–H]^−^ indicates that the mono-glutathione conjugate of PCA was present. Nevertheless, the LC-MS results revealed that a substantial signal of PCA (referred to as peak A) remains in its reduced form even after electrolysis. This phenomenon can be attributed to the presence of PCA that remains after the reaction, as well as interactions between the PCA quinone and glutathione. GSH acts by reducing the PCA quinone, effectively converting it back to its original form, PCA. As part of the investigation, products resulting from PCA electro-oxidation were also investigated when glutathione was present in the solution. In conducting the experiment, a potential of 0.3 V was kept constant and applied to the model wine solution containing PCA and GSH at a pH of 3.6 for 5 minutes. Then, the solution was analyzed using the UPLC-MS system. In the UPLC chromatogram of the PCA−GSH mixture, a distinct peak was detected at a wavelength of 295 nm (Figure 4A, represented by the red line, identified as peak C). This peak, labeled as peak C, corresponds to a di-glutathione conjugate of PCA mass, as indicated by its *m*/*z* value of 763 [M–2H]^2−^. Furthermore, the peaks observed in method 1, peaks A and B, were found to be identical to the peaks observed when PCA was electrochemically oxidized glutathione being present using method 2. This suggests that the two signals obtained in method 1 correspond to the same characteristic peaks observed in method 2. PCA di-glutathione conjugate formation is enhanced through the oxidation of the PCA-GSH mixture in the electrochemical cell.

The electro-oxidation behavior of CAF at pH 3.6 was also investigated. The electrolyzed solution was examined to determine if any electro-oxidation of CAF occurred at 0 V. No electrochemical oxidation of CAF was observed under these conditions. However, a peak corresponding to *m*/*z* 179 [M–H]^−^ was observed, which was identified as CAF. This is depicted in Figure 4B, where the black line represents peak A. The discovery of a new product was made when the electrolyzed solution was captured using glutathione at 0.22 V. This finding is depicted in Figure 4B, where the blue line represents peak B. A significant signal was observed at *m*/*z* 484 [M–H]^−^, corresponding to the CAF mono-glutathione conjugate. This observation suggests the formation of a CAF mono-glutathione conjugate under the given experimental conditions. The investigation focused on examining the products resulting from electrochemical oxidation. During the experiment, a constant potential of 0.22 V was applied to synthetic wine at pH 3.6 containing CAF and GSH for a duration of 5 min. Upon analysis, a distinct peak was obtained on the UPLC chromatogram of the GSH-CAF solution at 320 nm. This peak, labeled as peak C in Figure 4B (red line), indicated the presence of a new product. The mass peak labeled as Peak C at *m*/*z* 789 [M–2H]^2−^ indicates the presence of a CAF bi-glutathione conjugate. This conjugate is formed when CAF reacts with glutathione molecules. A similar mechanism has been proposed previously to explicate the occurrence of both mono- and bi-glutathione conjugates concerning hydroxycinnamic acids [52]. This mechanism suggests that the electrochemical oxidation of the CAF-GSH solution promotes the formation of CAF bi-glutathione conjugates. This enhanced formation of bi-glutathione conjugates may have important implications in understanding both CAF metabolism and its biological activity.

The electrochemical coulometric oxidation behavior of EC when GSH was present was investigated in synthetic wine (pH 3.6). The electrolyzed solution was analyzed to determine the extent of the electro-oxidation of EC. Interestingly, no EC electrochemical oxidation was observed at a potential of 0 V. Instead, just one peak corresponding to *m*/*z* 291 [M–H]^−^ was detected in the mass spectrum. This peak was attributed to EC and is represented by the black line in Figure 4C (peak A). A novel compound was identified when the electrolyzed solution was captured using GSH at 0.2 V (method 1). This observation is depicted in Figure 4C as a blue line, specifically at peak B. The corresponding *m*/*z* value of 596 [M–H]^−^ indicates the presence of the EC mono-glutathione conjugate. The investigation also explored the products resulting from the EC electro-oxidation reaction when glutathione was present in the solution. A constant potential of 0.2 V was applied to synthetic wine (pH 3.6) containing both EC and GSH for a duration of 5 min. Subsequently, these solutions were introduced in the UPLC-MS system for further analysis. The UPLC chromatogram showed a novel peak of the EC-GSH mixture when analyzed at a wavelength of 280 nm (refer to Figure 4C, red line, peak C). This peak exhibits a mass-to-charge ratio (m/0.5z) of 451 [M–H]^2−^. This particular *m*/*z* value corresponds to the molecular weight of a bi-glutathione conjugate of EC. In a paper that has been published [53], the authors detailed the discovery of different types of catechin glutathione conjugates, including mono-, bi-, and tri-glutathione conjugates.

The Ru electro-oxidation when glutathione was present was investigated using a consistent experimental procedure. First, Ru was not electrochemically oxidized at a potential of 0 V. Just one peak at *m*/*z* 611 [M–H]^−^ was observed corresponding to Ru. This observation is depicted in Figure 4D as a black line, specifically represented by peak A. At 0.3 V a recently discovered compound was identified when the electrolyzed solution was captured using GSH (method 1) (refer to Figure 4D, blue line, peak B). A significant signal at *m*/*z* 916 [M–H]^−^ was assigned to the Ru mono-glutathione conjugate. The investigation focused on analyzing the products resulting from the Ru electrochemical oxidation reaction when glutathione was present in the solution. To accomplish this, a constant potential of 0.3 V was applied to synthetic wine containing both Ru and GSH, maintained at a pH of 3.6. A novel peak was identified on the UPLC chromatogram at a wavelength of 355 nm (refer to Figure 4D, red line, peak C). The peak, with a mass-to-charge ratio (m/0.5z) of 611 [M–H]^2−^, was assigned to a Ru bi-glutathione conjugate. As a result, the combination of Ru and GSH undergoes electrochemical oxidation, which leads to an increased production of Ru-bi-glutathione conjugates.

**Scheme 2:**
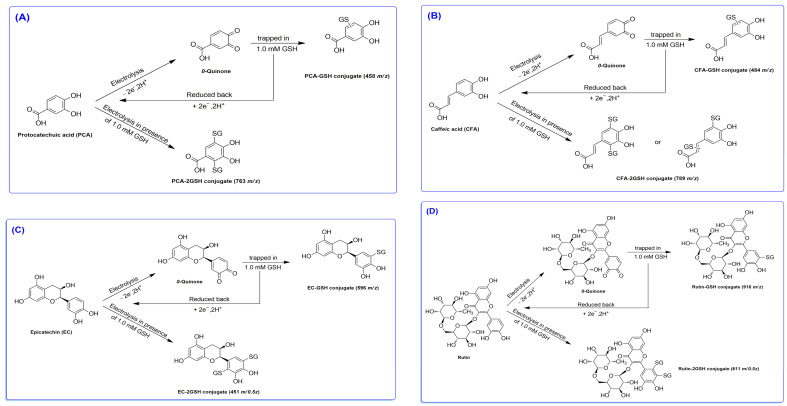
Proposed mechanism for (**A**) PCA, (**B**) CAF, (**C**) EC, and (**D**) Ru electrochemical oxidation when GSH is present.

Based on the aforementioned observations, Scheme 2 can be utilized to illustrate the electro-oxidation mechanisms of PCA, CAF, EC, and Ru. This comprehensive scheme encapsulates the intricate processes involved in the electrochemical oxidation of these compounds. By systematically presenting the mechanisms, Scheme 2 provides a clear and concise representation of the chemical transformations that occur during the oxidation of PCA, CAF, EC, and Ru. This scheme is a valuable tool for scientists seeking to comprehend and analyze the electrochemical behavior of these compounds. The presented findings indicate that polyphenols, upon undergoing electro-oxidation, generate an *o-*quinone intermediate. This intermediate readily combines with glutathione to form conjugates. Through mass spectrometry analysis, it was determined that mono- and bi-glutathione conjugates of PCA, CAF, EC, and Ru were formed, as illustrated in Scheme 2A–D. The polyphenols under investigation oxidation generates a quinone compound that exhibits remarkable reactivity. This quinone compound readily engages in reactions with nucleophilic molecules, including glutathione. Polyphenol radical species formation occurs through the induction of the catechol moiety by the loss of one electron and one proton. This process is initiated by specific signals. Additionally, the radical intermediate has the potential to submit a second electron–proton loss, resulting in quinone formation. These reactions are of main importance in the polyphenol’s chemical transformations. The presence of GSH nucleophiles facilitates the attack on different reactive species, including quinone. As a result of this interaction, polyphenols adduct species are formed. Specifically, when polyphenols are subjected to electrochemical oxidation, a quinone compound is generated. This quinone compound then submits a GSH addition reaction, bringing mono- and bi-glutathione conjugates.

## 4. Conclusions

In conclusion, wine polyphenols oxidation by electrochemistry in the presence of glutathione is a multifaceted process that encompasses various stages. It involves the transfer of electrons, the creation of reactive species, and subsequent reactions leading to the formation of oxidized products. The characterization of this intricate process is of main interest for the determination of wines’ sensory characteristics, making it imperative for winemakers to comprehend and effectively control them. Indeed, the formation of oxidized products can significantly impact the taste, color, and aroma of wine, ultimately influencing the overall quality and consumer perception. Through careful control of the oxidation process with glutathione, winemakers can maintain the preservation of compounds of interest in white wine. Furthermore, understanding the complex interactions between wine polyphenols, glutathione, and the electrochemical environment enables winemakers to optimize the use of antioxidants and other additives.

## Data Availability

All of the data is contained within the article.
